# Infrared and visible image fusion algorithm based on spatial domain and image features

**DOI:** 10.1371/journal.pone.0278055

**Published:** 2022-12-30

**Authors:** Liangjun Zhao, Yun Zhang, Linlu Dong, Fengling Zheng

**Affiliations:** 1 Computer Science and Engineering, Sichuan University of Science and Engineering, Yibin, Sichuan, China; 2 Grassland Research Institute, Xinjiang Academy of Animal Sciences, Urumqi, Xinjiang, China; Sant Longowal Institute of Engineering and Technology, INDIA

## Abstract

Multi-scale image decomposition is crucial for image fusion, extracting prominent feature textures from infrared and visible light images to obtain clear fused images with more textures. This paper proposes a fusion method of infrared and visible light images based on spatial domain and image features to obtain high-resolution and texture-rich images. First, an efficient hierarchical image clustering algorithm based on superpixel fast pixel clustering directly performs multi-scale decomposition of each source image in the spatial domain and obtains high-frequency, medium-frequency, and low-frequency layers to extract the maximum and minimum values of each source image combined images. Then, using the attribute parameters of each layer as fusion weights, high-definition fusion images are through adaptive feature fusion. Besides, the proposed algorithm performs multi-scale decomposition of the image in the spatial frequency domain to solve the information loss problem caused by the conversion process between the spatial frequency and frequency domains in the traditional extraction of image features in the frequency domain. Eight image quality indicators are compared with other fusion algorithms. Experimental results show that this method outperforms other comparative methods in both subjective and objective measures. Furthermore, the algorithm has high definition and rich textures.

## Introduction

Although a huge amount of sensor data has been collected with the advancement of sensor technology, single sensor data can provide limited information about the scene. Thus, multiple sensors have recently been combined to obtain more comprehensive and accurate scene information. However, the different characteristics of different sensors cause some challenges in subsequent processes. Also, multiple sensors provide redundancy in the collection of information, resulting in low utilization of transmission bandwidth and storage space. In order to overcome these problems, image fusion technology has been researched in many fields. Especially, the fusion of the visible-light image and infrared image has attracted attention due to its practical value in the military [1, 2] and security applications [3]. Infrared images are sensitive to the heat source targets in the scene, enabling users to quickly grasp the target location information. However, the infrared sensor cannot detect cold or heat-equilibrium objects in practical applications, losing much useful information [4]. In contrast, the visible-light sensor can provide an image with rich scene texture, but the collected image is susceptible to the influence of light intensity and smoke occlusion. It often makes the target information difficult to be quickly recognized by the user [5]. Therefore, the fusion of infrared image and visible-light image allows combining the advantages of each source image into one image.

Image fusion algorithms are divided into three groups according to the fusion level: pixel level, feature level, and decision level fusions [6]. Further, a hybrid level fusion has been explored to combine different levels of fusion methods. In the past few decades, many classic image fusion algorithms have emerged, such as deep learning-based methods [7, 8], neural network-based methods [1], sparse representation-based methods [2], and subspace-based methods [3]. However, those methods provided insufficient image anti-interference ability with a large amount of information loss in the infrared images. Multi-scale decomposition [6] was proposed to zoom the image and superimpose multiple layers of spatial feature information for the effective fusion of infrared images and visible-light images, based on multi-scale transform, including pyramid transform [7], Wavelet transform [8], curvelet transform [9], contourlet transform [10], and non-subsampled contourlet transform [11]. However, regardless of the effectiveness of multi-scale decomposition, the fused images often suffered from halos artifacts and other interference factors [12]. In addition, determining and decomposing the number of layers in the image space is a challenge. The larger the number of decomposition layers, the richer the texture details of the fused image. However, the middle- and low-frequency layer coefficients affect most fused pixel values in the fused image as the number of image decomposition layers increases. The fusion effect and the robustness of the algorithm are a trade-off. Accordingly, a single-level fusion approach is difficult to deal with complex and diverse image fusion problems.

**Fig 1:**
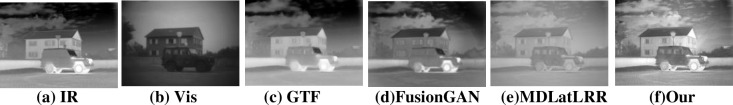
Schematic illustration of image fusion. (a) IR, (b) Vis, (c) GTF, (d) FusionGAN, (e) MDLatLRR, (f) Our.

With the development of machine learning technology methods such as neural networks, scholars introduced support vector machines and genetic algorithms into image processing [13–21] to solve the defects of traditional frequency-domain feature extraction technology. Among them, the neural network method is more common in image fusion, which includes three types. The first is based on pre-training the network. This network model allows the machine to extract images adaptively through many pre-training models. However, this method requires large-scale training samples. The extracted features cannot meet the application requirements under insufficient samples. At the same time, the neural network models are designed for classifiers and may not be suitable for image fusion tasks. In the second type proposed by Li et al., the self-encoding network was introduced into image fusion. The network comprises an encoder and a decoder. The encoder can extract the features of the image to be fused, and the decoder generates the fusion image, which solves the tuning requirement problem. However, both the encoder and the decoder should be designed for the corresponding fusion task, and the algorithm has weak robustness. In the third type, many scholars proposed neural networks to solve the defects of fused images. Jiayi Ma proposed FusinGAN [22]. With a well-designed network structure, good fusion results can be achieved without hand-crafted strategies. However, a vast amount of data specified to the target fusion tasks are required, which often cannot perform well for the different data. For example, the fusion model trained with bright images cannot work well for low illumination images. Hui Li [23] proposed a novel image fusion framework based on MDLatLRR, where the source image was decomposed into detail parts (salient features) and base parts. Then, those detail and base parts were fused by the nuclear-norm-based fusion strategy. This method effectively solved the artifacts and halo that happened in the traditional multi-scale fusion method (Fig 1). Still, the integral definition of the fused image remains to be optimized. The MDLatLRR is similar to the anisotropic diffusion-based method. Both methods decomposed the infrared and visible light images into the basic and detail layers, although the decomposition algorithm and fusion rules are different. They can prevent artifacts generated in the fusion process, but both suffer poor image definition.

In order to overcome the above-mentioned issues, this paper proposes an infrared and visible image fusion method based on spatial domain and image features. In the method, an efficient multi-scale decomposition is adopted to smooth the source image, which can effectively extract the intermediate frequency layers of the infrared image and the visible-light image. Unlike strong decomposition algorithms such as the anisotropic diffusion algorithm [24], the proposed multi-scale decomposition rather causes large edge gradients between different pixel groups of the intermediate frequency layer. Thus, the mean filter is applied to obtain a better intermediate frequency layer. Also, the high-frequency and low-frequency layers are extracted, and then the large layer and the small layer of each source image are extracted. The final fused image is obtained through the adaptive weight fusion according to the feature parameters of each source image and layer pixel.

To highlight the major advantage of our method, we illustrate a representative example in Fig 1. The infrared and visible images are fused, where the visible image contains detailed background and the infrared image highlights the target, i.e., the pedestrian. Fig 1C and 1D show the fused images by the traditional method (GTF) and advanced method (FusionGAN), respectively. Although it highlights the target location information, the clarity of the environmental texture display is not significantly improved. The fused images by MDLatLRR (Fig 1E) can reflect the information of the infrared and visible images, but it is still blurred. In contrast, our method (Fig 1F) can provide a fused image where environment texture and target information can be easily recognized.

The experimental results show that the proposed fusion method provides a higher definition and more comprehensive information than the other fusion algorithms. The contributions of this paper are as follows. First, we propose an image clustering algorithm for image fusion, greatly improving the quality of fused images. Second, different from the traditional multi-scale decomposition strategy, we extract one more dark detail layer on the basis of predecessors, which makes the texture gradient of the fusion result easier to reflect the information carried by the image. As a result, the clarity of the fused image is improved. Third, we propose a fusion method based on the principle of expanding texture and the layer eigenvalue as the weight. This makes the weight-average strategy more effective, addressing the poor texture definition of existing fused images.

The remainder of this paper is structured as follows. Section 2 describes the proposed method in detail. Section 3 introduces the parameter settings of the proposed fusion theory. Section 4 provides experimental results, and Section 5 concludes this paper.

## Proposed method

This section describes the proposed method in detail, followed by the analysis of the parameter setting. The proposed method first decomposes images in multi-scale by using the superpixel-based fast fuzzy C-means. Then, the high-frequency layer and low-frequency layer of the source image are extracted. Lastly, those layers are combined with a fusion strategy to obtain the final fused image.

### A. Mathematical model

**Fig 2:**
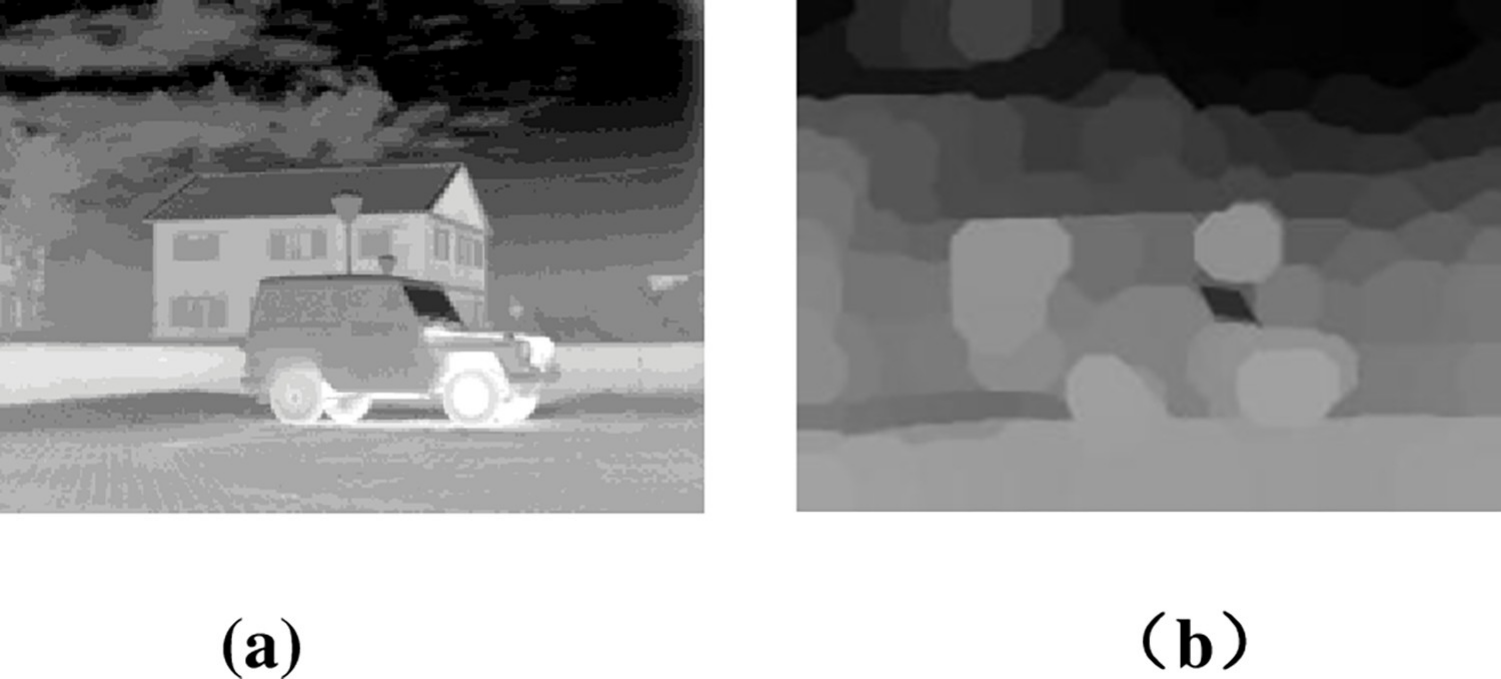
Example of the multi-scale decomposition (a) source image, (b) the proposed multi-scale decomposition.

The multi-scale decomposition for image fusion requires the following two conditions. 1) it can reasonably and effectively decompose the source image information in multi-scales, and 2) the computational time should be fast. Accordingly, the multi-scale decomposition method is designed to achieve multi-scale decomposition with a low computational time. We propose the superpixel-based fast fuzzy C-means image hierarchical multi-scale decomposition method. The decomposition effect of the source image is shown in Fig 2.

Fig 2 shows the schematic diagram of the proposed multi-scale decomposition method to extract the intermediate frequency layer. In the intermediate frequency layer, the clustered areas of the source image are effectively retained, and the texture gradient between the segments is weakened. However, the clustering results (Fig 2B) still have a lot of texture information. Therefore, we need to improve the algorithm to fully extract texture information.

**Fig 3:**
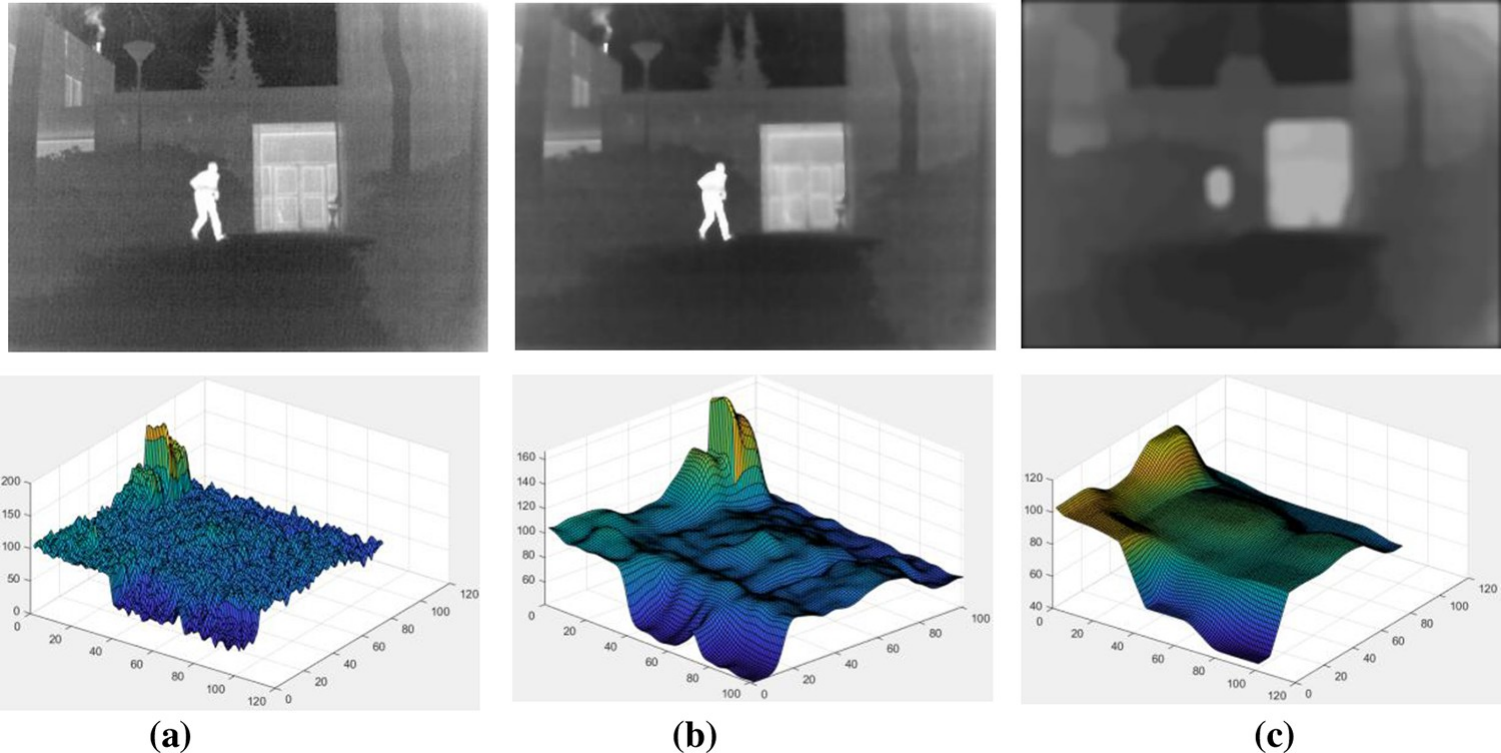
A surf plot of the proposed clustering algorithm versus ADF clustering results. The first row includes, from left to right: infrared image, ADF clustering results, and the proposed algorithm clustering results; the second row includes, from left to right: infrared image partial area surf map, ADF clustering partial area surf map, and the proposed clustering algorithm partial area surf map. The abscissa of the Surf map represents the spatial position of the pixel, and the ordinate represents the size of the pixel value.

In order to evaluate the image clustering ability of the proposed clustering algorithm, the image clustering algorithm in [24] is employed for comparison, and the resulting surf graph is shown in Fig 3.

As shown in Fig 3, the proposed clustering algorithm has a higher degree of image blurring than ADF. Combined with the surf map observation, the surf map corresponding to the infrared image contains many texture gradients. Although the surf map of ADF smooths a lot of details texture, there is still a lot of nonsmoothed information compared with our surf map, which will lead to multi-scale decomposition. Accordingly, our fusion results are more precise since part of the information in the feature layer cannot be extracted.

#### 1) Mathematical model of the proposed multi-scale decomposition

According to the above multi-scale decomposition tool conditions, the existing image clustering algorithms, such as the C-means clustering algorithm and K-means clustering algorithm, are analyzed while developing the multi-scale decomposition tool. The iterative calculation of distances between pixels increases the computational cost of the clustering algorithm. At the same time, the local features are destroyed due to the fixed dimension of the clustering window, making the clustering result unfavorable for subsequent multi-scale decomposition. Therefore, image clustering is implemented by adopting a filter window based on c-means with better adaptive and irregular local space provided by superpixels, which can effectively solve the problems of high computational time cost and local structure damage during clustering.

The objective function of the proposed multi-scale decomposition is defined as follows. The determination of the multipliers is given by [25]:

$$J_{m}=\sum_{l=1}^{q}\sum_{k=1}^{c}S_{l}u_{kl}^{m}{\left\|(\frac{1}{S_{l}}\sum_{p\in R_{l}}x_{p})-u_{k}\right\|}^{2}$$ (1)

where l represents the gray intensity levels, q is the number of superpixels, and 1≤l≤q. *S*_*l*_ represents the number of pixels in the lth region *R*_*l*_, and c indicates the number of image clusters. *u*_*kl*_ is the membership matrix of the area of the lth superpixel, and the *u*_*k*_ represents the center of the kth cluster. ***x***_***p***_ represents a pixel value in the image. c is the number of clusters.

The optimization problem of Formula (1) can be transformed into an unconstrained problem using a Lagrangian multiplier, thereby minimizing the objective function and satisfying:

$$\tilde{J}m=\sum_{l=1}^{q}\sum_{k=1}^{c}S_{l}u_{kl}^{m}{\left\|(\frac{1}{S_{l}}\sum_{p\in R_{l}}x_{p})-u_{k}\right\|}^{2}-\lambda (\sum_{k=1}^{c}u_{kl}-1)$$ (2)

where *λ* is the Lagrangian multiplier. The *u*_*kl*_ and *u*_*k*_ partial differential equation in Eq (2) satisfies:

$$\begin{array}{l} \frac{\partial {\tilde{J}}_{m}}{\partial u_{kl}}=\sum_{l=1}^{q}\sum_{k=1}^{c}\frac{\partial S_{l}u_{kl}^{m}{\left\|(\frac{1}{S_{l}}\sum_{p\in R_{l}}x_{p})-u_{k}\right\|}^{2}}{\partial u_{kl}}-\lambda \\ \;={\sum_{l=1}^{q}\sum_{k=1}^{c}mS_{l}u_{kl}^{m-1}\|(\frac{1}{S_{l}}\sum_{p\in R_{l}}x_{p})-u_{k}\|}^{2}-\lambda \\ \;=0 \end{array}$$ (3)

$$\begin{array}{l} \frac{\partial {\tilde{J}}_{m}}{\partial u_{k}}=\sum_{l=1}^{q}\sum_{k=1}^{c}\frac{\partial S_{l}u_{kl}^{m}{\left\|(\frac{1}{S_{l}}\sum_{p\in R_{l}}x_{p})-u_{k}\right\|}^{2}}{\partial u_{kl}}\\ \;=\sum_{l=1}^{q}\sum_{k=1}^{c}S_{l}u_{kl}^{m}\frac{\partial {\left\|(\frac{1}{S_{l}}\sum_{p\in R_{l}}x_{p})-u_{k}\right\|}^{2}}{\partial u_{k}}\\ \;=\sum_{l=1}^{q}S_{l}u_{kl}^{m}\frac{\partial {\left\|(\frac{1}{S_{l}}\sum_{p\in R_{l}}x_{p})-u_{k}\right\|}^{2}}{\partial u_{k}}\\ \;=-2\sum_{l=1}^{q}S_{l}u_{kl}^{m}\|\frac{1}{S_{l}}\sum_{p\in R_{l}}x_{p}-u_{k}\|\\ \;=0 \end{array}$$ (4)

Combining the Formulas (3) and (4), *u*_*kl*_ and *u*_*k*_ are computed as follows:

$$u_{k}=\frac{\sum_{l=1}^{q}u_{kl}^{m}\sum_{p\in R_{l}}x_{p}}{\sum_{l=1}^{q}S_{l}u_{kl}^{m}}$$ (5)

$$u_{kl}=\frac{{\left\|(\frac{1}{S_{l}}\sum_{p\in R_{l}}x_{p})-u_{k}\right\|}^{-2/(m-1)}}{\sum_{j=1}^{c}{\left\|(\frac{1}{S_{l}}\sum_{p\in R_{l}}x_{p})-u_{j}\right\|}^{-2/(m-1)}}$$ (6)

The process of decomposition is conducted as follows:

(A) The same pixels of the source image are combined according to the function *J*_*m*_, denoted as *I* (*x*).
(B) Morphological expansion processing is performed. *I* (*x*) and b represent the morphological window dimension and the window dimension value, respectively. The result after the expansion is denoted as *I*_1_ (*x*), where *I*_1_(*x*) = *I*(*x*)⊕*b* (Fig 6).
(C) Morphological corrosion treatment is performed. The result after the expansion is denoted by *I*_2_ (*x*), where *I*_2_ (*x*) = *I*_1_(x) ⊝*b*. Therefore, we have:
$$I_{2}(x)=(I_{1}(x)⊕b)⊖b$$ (7)
(D) The processed layers are composed into an image *I*_2_ (*x*), followed by the mean filtering with a *m*×*m* window to obtain the final rsults, denoted as *I*_*M*_.

#### 2) Extraction of high-frequency and low-frequency layers

The multi-scale decomposed image *I*_*M*_ is obtained with the window *W*_*n*×*n*_ where n = 150 as the intermediate frequency layer. The high-frequency layer *I*_*H*_ is obtained by subtracting the intermediate frequency layer *I*_*M*_ from the source image *I*_*S*_:

$$I_{H}=I_{S}-I_{M}$$ (8)

The low-frequency layer image *I*_*L*_ is obtained by subtracting the source image *I*_*E*_ from the intermediate frequency layer *I*_*M*_:

$$I_{L}=I_{M}-I_{S}$$ (9)

**Fig 4:**
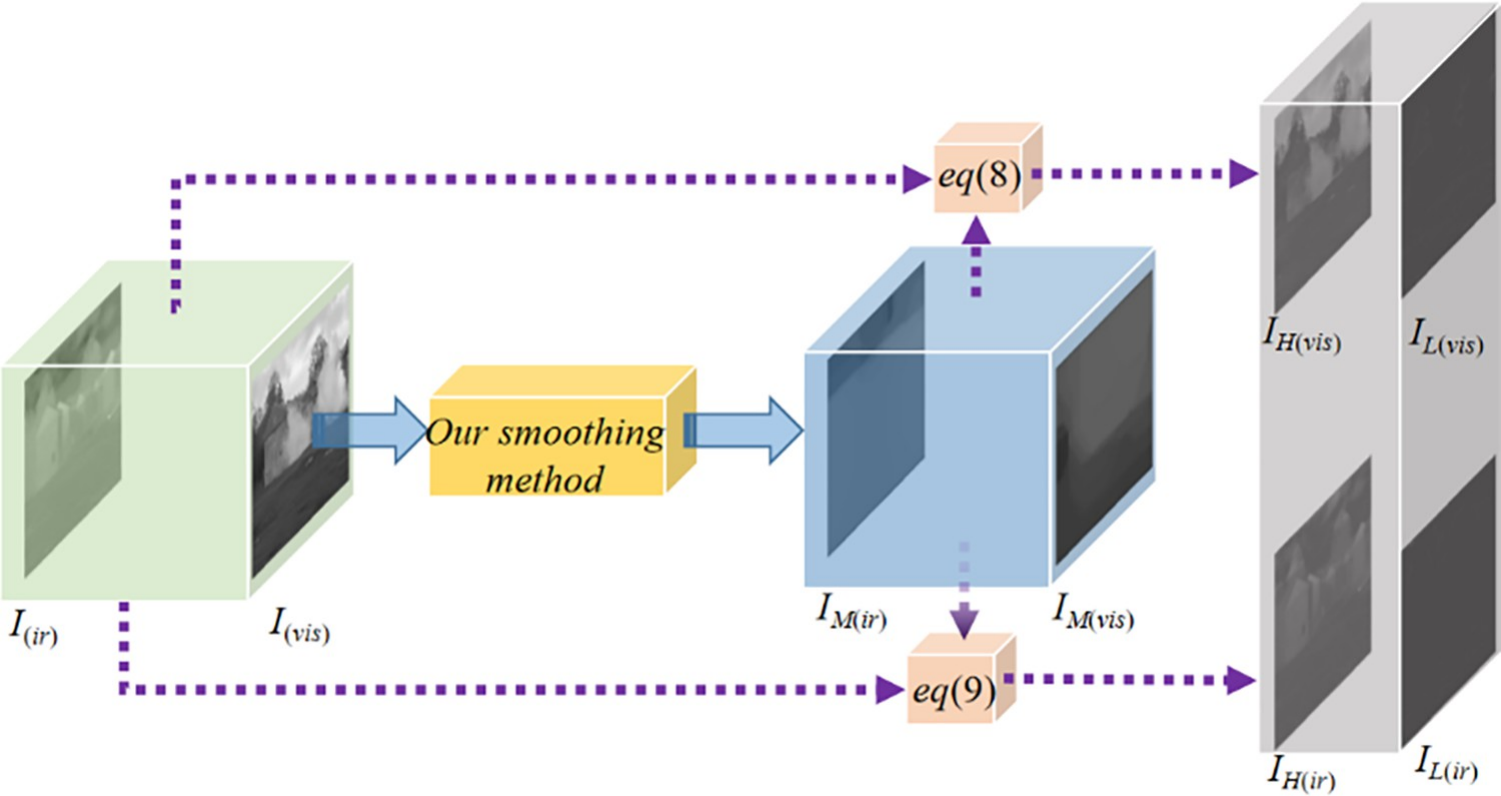
Multi-scale decomposed images for visible-light and infrared images. I_(ir)_ represents the infrared image, I_(vis)_ represents the visible image, and the yellow cube represents the proposed smoothing method. The processed infrared and visible images are denoted as I_M(ir)_ and I_M(vis)_, respectively. Through Eq (8), the visible image is decomposed into bright detail layer I_H(vis)_ and dark detail layer I_L(vis)_, while, through Eq (9), the infrared image is decomposed into bright detail layer I_H(ir)_ and dark detail layer I_L(ir)_.

The multi-scale decomposition results of the source image are shown in Fig 4.

### B. Fusion strategy

The fusion process consists of two parts:

1) intermediate-frequency layer fusion and 2) high-frequency and low-frequency layers fusion. First, in the fusion process of the intermediate-frequency layer, the pixels with the larger value between visible-light and infrared images are selected for the maximum layer. The pixel with the smaller value is selected at the corresponding coordinates of the two images to form the minimum layer.

Then, the fused image is computed as follows:

$$\begin{array}{l} I_{Z}=\frac{\min(\sigma_{1},\sigma_{2})}{\sigma_{1}+\sigma_{2}}\max(I_{M1},I_{M2})+\\ \;\frac{\max(\sigma_{1},\sigma_{2})}{\sigma_{1}+\sigma_{2}}\min(I_{M1},I_{M2}) \end{array}$$ (10)

where *I*_*Z*_ indicates the fusion result of the frequency images in each source image. *I*_*M***1**_ and *I*_*M***2**_ represent the intermediate frequency layers of the visible-light and infrared images. max(*I*_*M***1**_,*I*_*M***2**_) represents the pixel standard deviation corresponding to the extremely large layer of the intermediate frequency image expressed by σ_1_, and min(*I*_*M***1**_,*I*_*M***2**_) represents the pixel standard deviation corresponding to the extremely small layer of the intermediate frequency image expressed by *σ*_2_.

The existing multi-scale algorithm converts the image from the spatial domain into the frequency domain and extracts the image features in the frequency domain using the corresponding fusion strategy, such as orthogonal change, sparse representation, and other methods. In order to avoid information loss and increase the amount of computation caused by the transformation between the frequency and spatial domains, the image features in the spatial domain are directly extracted, and the multi-scale decomposition method is applied to linear addition and subtraction. The fusion strategy also linearly reconstructs the fused image. The target is not outlined, although *I*_*Z*_ retained the basic texture of the source image. In order to compensate for this limitation, the maximum mixed image max(*I*_*S***1**_,*I*_*S***2**_) and the minimum mixed image min(*I*_*S***1**_,*I*_*S***2**_) of the source image are used. *I*_*S***1**_ and *I*_*S***2**_ represent the visible-light and infrared source images, respectively. *max*(*I*_*E*_, *I*_*I*_) and *min*(*I*_*E*_, *I*_*I*_) are respectively linearly fused with the intermediate frequency layer *I*_*M*_ with a weight of 0.1. The fused image *I*_*ZF*_ of the new intermediate frequency layer after the texture enhancement is formulated as follows:

$$I_{ZF}=I_{Z}+\frac{max(I_{S1},I_{S2})}{min(I_{S1},I_{S2})}\times (\max\left(I_{S1},I_{S2}\right)-min(I_{S1},I_{S2}))$$ (11)

**Fig 5:**
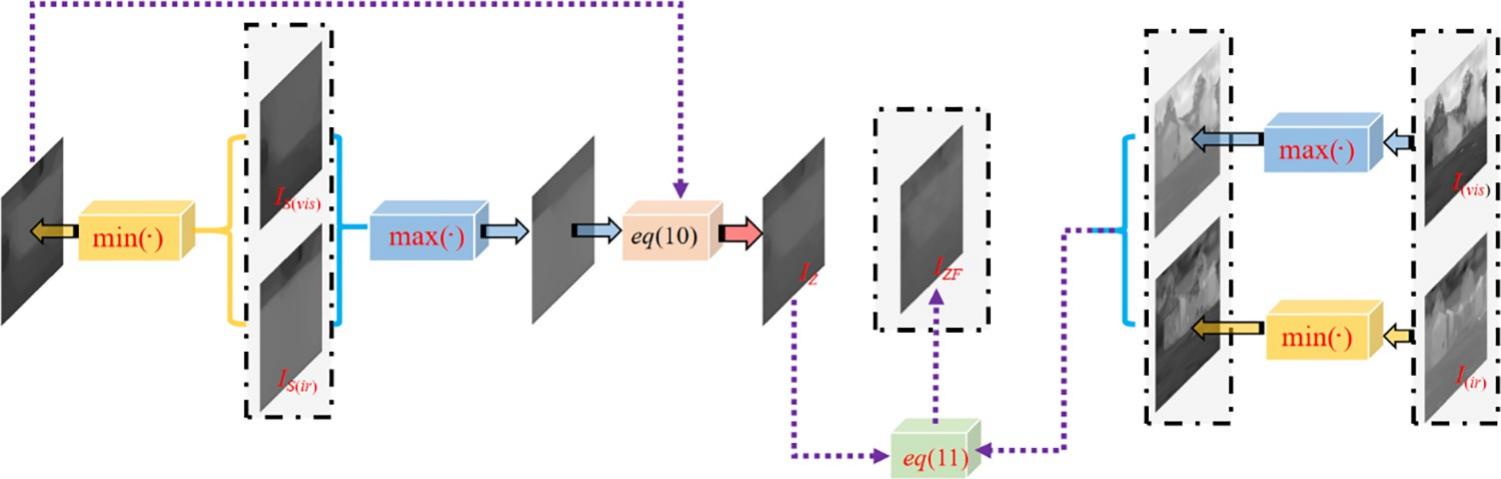
Formulation process of I_Z_ at intermediate frequency layer.

The formulation process of I_Z_ at the intermediate frequency layer is shown in Fig 5.

In the second part of the fusion process, the high-frequency and low-frequency layers are fused based on the fused intermediate layer *I*_*ZF*_ under the principle of texture gradient expansion. It is defined as follows:

$$I_{F}=I_{ZF}+\omega_{1}\times I_{H1}+\omega_{2}\times I_{H2}-\omega_{1}\times I_{L1}-\omega_{2}\times I_{L2}$$ (12)

Where *I*_*F*_ represents the fused image. *I*_*H***1**_ and *I*_*L***1**_ are the high-frequency layer and low-frequency layer of the visible-light image, respectively. *I*_*H***2**_ and *I*_*L***2**_ are the infrared image’s high-frequency and low-frequency layers, respectively. Inspired by the existing linear fusion algorithm, the total weight of linear fusion is 1, and the fusion weight of each base layer is 0.5, such as the basic fusion strategy of the ADF algorithm. We believe that the fixed fusion weight cannot provide strong robustness to the algorithm. Therefore, since the total weight is 1 and the fusion weight of each layer is 0.5, the weights are adjusted appropriately according to the feature ratio of the layers to be fused to improve the robustness of the fusion algorithm. *ω*_1_ and *ω*_2_ respectively represent the fusion weights controlling the relative significance of the visible-light and infrared high-frequency layers, defined as follows:

$$W=1+\frac{\left(\sqrt{\sigma_{3}+\sigma_{4}}-\sqrt{\sigma_{1}+\sigma_{2}}\right)}{\sqrt{\sigma_{1}+\sigma_{2}}}$$ (13)

$$\omega_{1}=W\times (0.5+\frac{\sigma_{5}-\sigma_{6}}{\sigma_{3}+\sigma_{4}})$$ (14)

$$\omega_{2}=W\times (0.5+\frac{\sigma_{6}-\sigma_{5}}{\sigma_{3}+\sigma_{4}})$$ (15)

where *W* indicates the sum of the weights of the high-frequency detail layer fusion of each source image. *σ*_**3**_, *σ*_**4**_, *σ*_**5**_, and *σ*_**6**_ represent the standard deviations of visible-light image *I*_*S***1**_, infrared image *I*_*S***2**_, the visible-light intermediate frequency layer *I*_*M***1**_, and the visible-light intermediate frequency layer *I*_*M***2**_, respectively.

**Fig 6:**
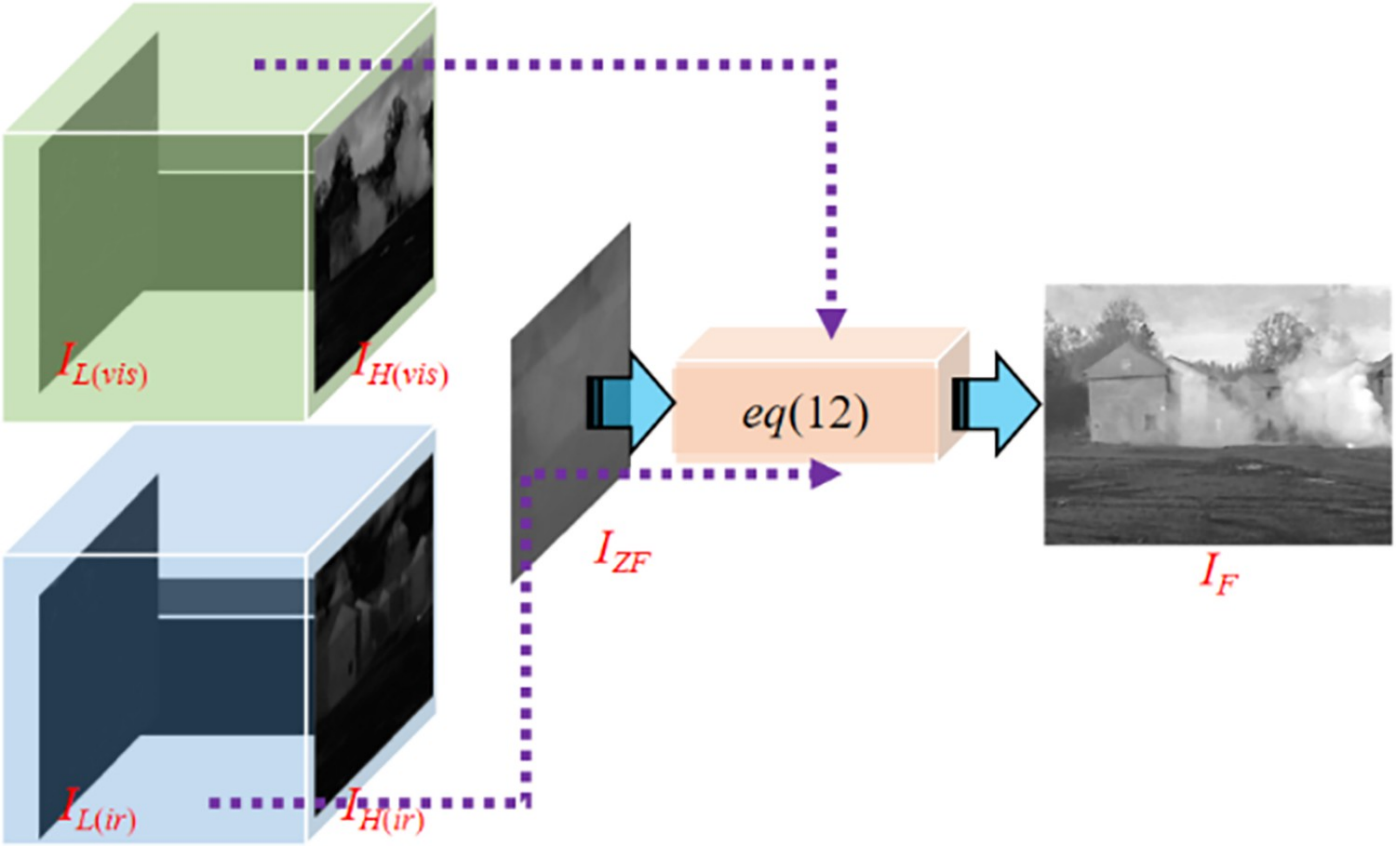
The fusion process to obtain the final result I_F_.

In combination with intermediate frequency layer I_Z_, high- and low-frequency layers of infrared ad visible light layers are fused into the final fusion result I_F_, as shown in Fig 6.

## Parameter settings

The proposed algorithm includes several parameters required to be set. In this Section, the influence of the parameters on the results is analyzed to determine the optimal settings.

### A. The dimension of the clustering window

**Fig 7:**
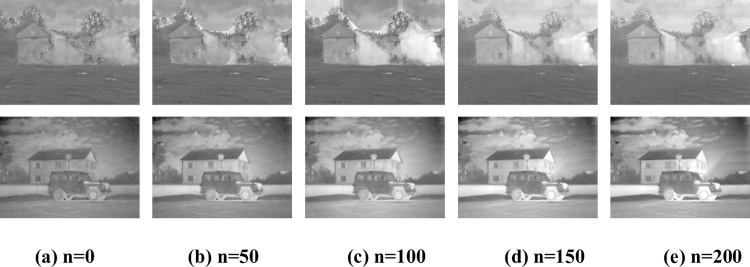
Fusion results according to the clustering window dimension. (a) n = 0, (b) n = 50, (c) n = 100, (d) n = 150, (e) n = 200.

The clustering results *I*_*C*_ are varied according to the clustering window dimension n, affecting the frequency layers extraction and, consequently, the image fusion results. Fig 7 depicts the fusion results according to the clustering window dimension n. As shown in Fig 7, when n is 0, many scattered artifacts appear in the fusion result. As the dimension n increases, such artifacts gradually gather to form a concentrated artifact area. Also, the overall brightness and sharpness of the image gradually increase until n = 150, while most artifacts are eliminated. When n = 200, the effect of the "smoke" image is almost the same as that of n = 150, and the artifacts of the "military car" become more severe. Accordingly, the window dimension n is set to 150.

### B. The dimension of the mean filter window

**Fig 8:**
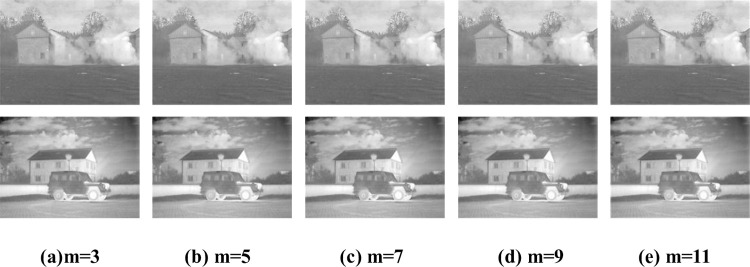
Fusion results according to mean filter window dimension. (a)m = 3, (b) m = 5, (c) m = 7, (d) m = 9, (e) m = 11.

In this Section, the mean filtering result, applied to remove the artifacts in *I*_*C*_, is analyzed according to the window dimension m. The fusion results according to a varied dimension m are shown in Fig 8 as the dimension of the mean filter window *W*_*m*×*m*_ increases, the artifacts are removed more. When m = 11, the obtained fusion result is almost the same as in the ’smoke’ image when m = 11. There is a slight difference in the ’Military Vehicles’, and the fusion effect when m = 11 is better than when m = 9. Thus, the window dimension m is set to 11.

The flow chart of the proposed method is given in Table 1.

**Table 1:** The flow chart of the proposed algorithm.

Infrared and visible image fusion based on spatial domain and image features
Input: Visible image I_V_, Infrared image I_I_.
Output: Fusion image I_F_.
1. The superpixel fast pixel clustering method proposed in this paper is used to process the source map respectively to obtain the source map base map I_M1_、I_M2_。
2. Then, the respective high-frequency image and low-frequency image are proposed through Eqs (8) and (9) and various intermediate frequency layers I_M1_、I_M2_.
3. The base level image of each source image is fused by Eq (10) to obtain the base level fusion image I_Z_.
4. Eq (11) enhances the texture of the base image I_Z_ to obtain I_ZF_.
5. Fuse the high-frequency image and low-frequency image of each source image obtained in step 3 with I_Z_ according to Eq (12) to obtain the final fusion result I_F_.

**Fig 9:**
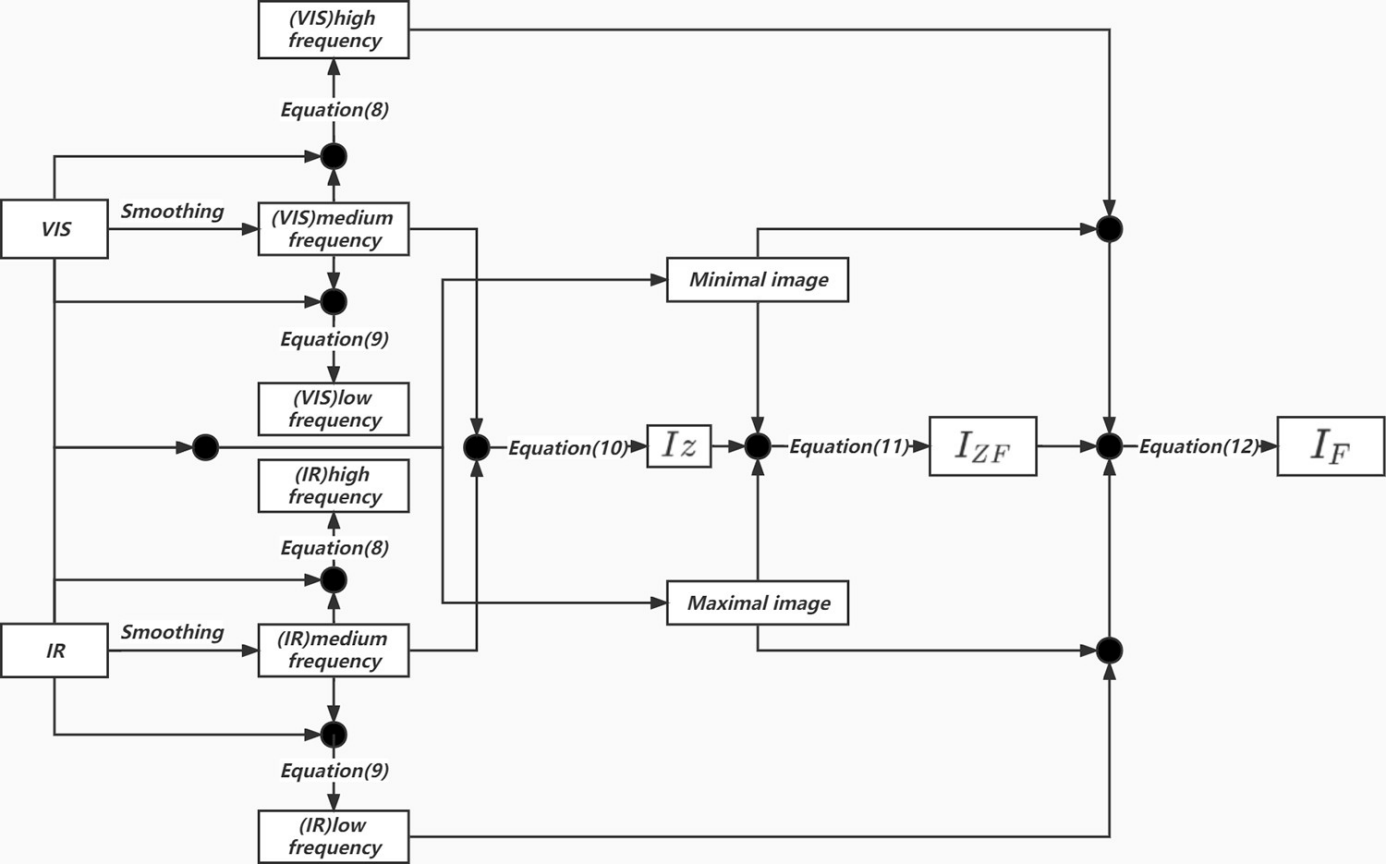
The diagram of the proposed algorithm.

It is worth noting that the most time-consuming part of the proposed algorithm is step 2: the image multi-scale decomposition. The diagram of the proposed algorithm is depicted in Fig 9.

The computational complexity of the proposed method mainly includes the following categories:

1. Multi-scale decomposition includes image clustering and image smoothing. Therefore, its computational complexity is *O*(*n*^2^).
2. The computational complexity of image reconstruction for each layer is *O*(*n*)
3. The final complexity T(n) of the proposed method is expressed as follows:

$$T(n)\to O(n^{2})+O(n)+O(n)$$ (16)

## Simulation results

The performance of the proposed method is validated in terms of both objective and subjective evaluations, compared with the comparison algorithms, including Nestfuse [26], FusionGAN [27], MDLatLRR [28], SEDRFuse [29], STDFusionNet, GANMcC [22], and ResNetFusion [30]. All experiments were conducted on Windows 10, 2.60GHz CPU, 8GB RAM, with MATLAB2016a. The experimental data were obtained from: TNO_Image_Fusion_Dataset(https://figshare.com/articles/dataset/TNO_Image_Fusion_Dataset/1008029).

### A. Evaluation metrics

The objective evaluation indexes include average gradient (AG) [31], information entropy (H) [32], standard deviation (SD) [33], spatial frequency (SF) [34], edge strength (EI) [35], fusion function (Q^***ab/f***^) [36], the amount of artifact (N^***ab/f***^) [37], and the fusion loss function (L^***ab/f***^) [33]. The larger the evaluation value of AG, H, SD, SF, and EI, the better the image quality, the larger the Q^***ab/f***^ value indicates that the fusion image contains more information of the source image, and the smaller the N^***ab/f***^ value indicates that the fusion image is produced, the fewer artifacts. The smaller the L^***ab/f***^ value, the smaller the loss of source image information during the fusion process.

### B. Evaluation of fusion performance

#### 1) Dataset

The performance of the compared methods was evaluated on the surveillance images from TNO Human Factors. The dataset includes registered multispectral night-time imagery of different military-relevant scenarios. We selected seven typical pairs for qualitative illustration: Two men in front of the house, Soldierbehindsmoke_3, Soldierintrench_1, Houseswith3men, Kaptein_1123, Marne_04, and Sandpath. In addition, we tested our method on the INO database, which is provided by the National Optics Institute of Canada and contains several pairs of visible and infrared videos representing different scenarios captured under various weather conditions. Specifically, we grabbed 21 visible and infrared image pairs from the video named Trees and runner for qualitative and quantitative comparisons.

#### 2) Results of the TNO dataset

**Fig 10:**
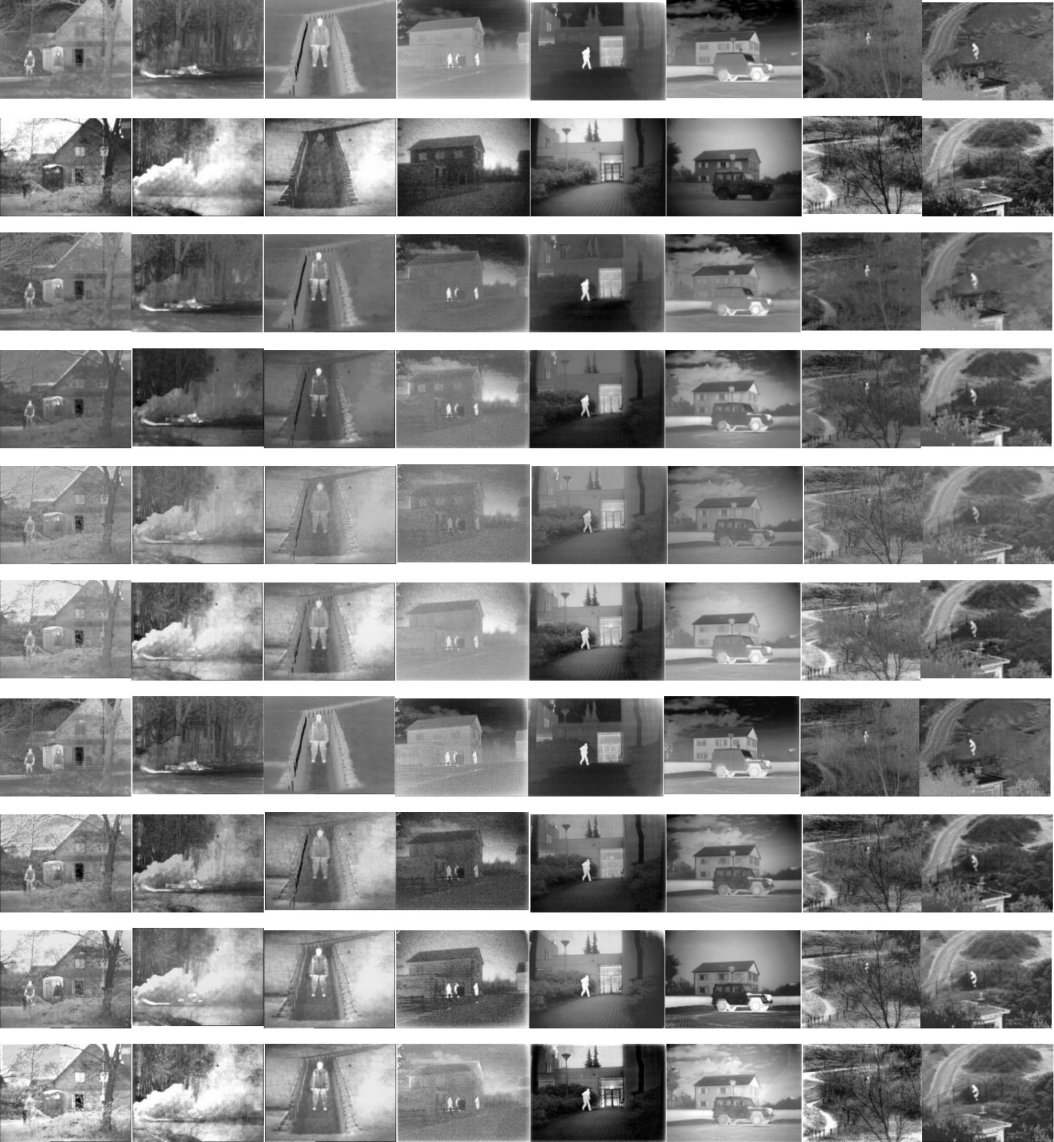
Qualitative fusion results for eight typical infrared and visible image pairs from the TNO database. (The first row: Infrared images; The second row: Visible images; The third row: FusionGAN; The fourth row: GANMcC; The fifth row: MDLatLRR; The sixth row: NestFuse; The seventh row: RESNetFusion; The eighth row: SEDRFuse; The ninth row: STDFusionNet; The tenth row: OUR).

Eight typical image pairs from the TNO dataset were used to qualitatively evaluate the performance of the proposed method and the compared seven methods, as shown in Fig 10.

**Fig 11:**
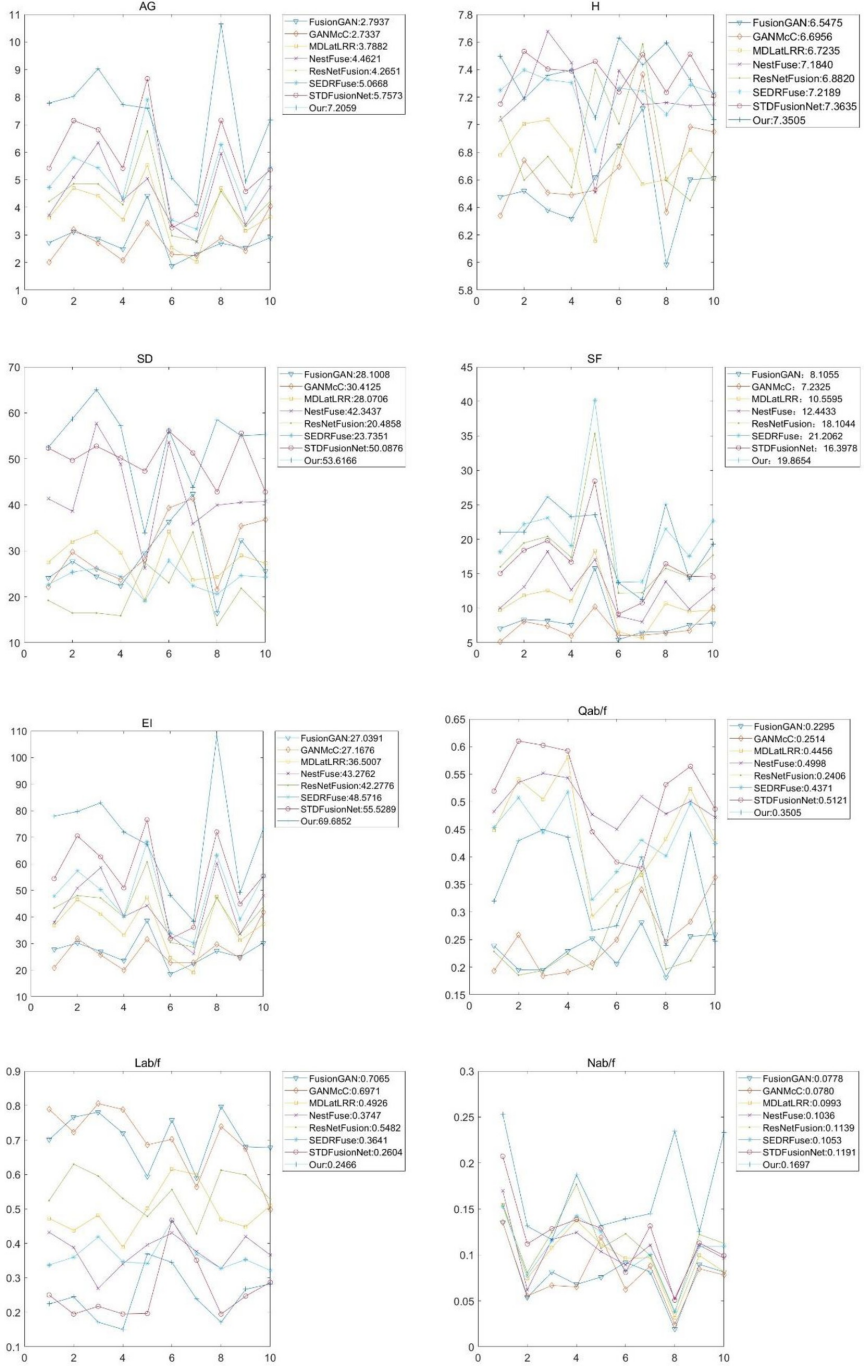
Quantitative comparisons in terms of the eight metrics: AG, H, SD, SF, EI, Q^ab/f^, L^ab/f^, and N^ab/f^, for ten image pairs from the TNO database.

**Fig 12:**
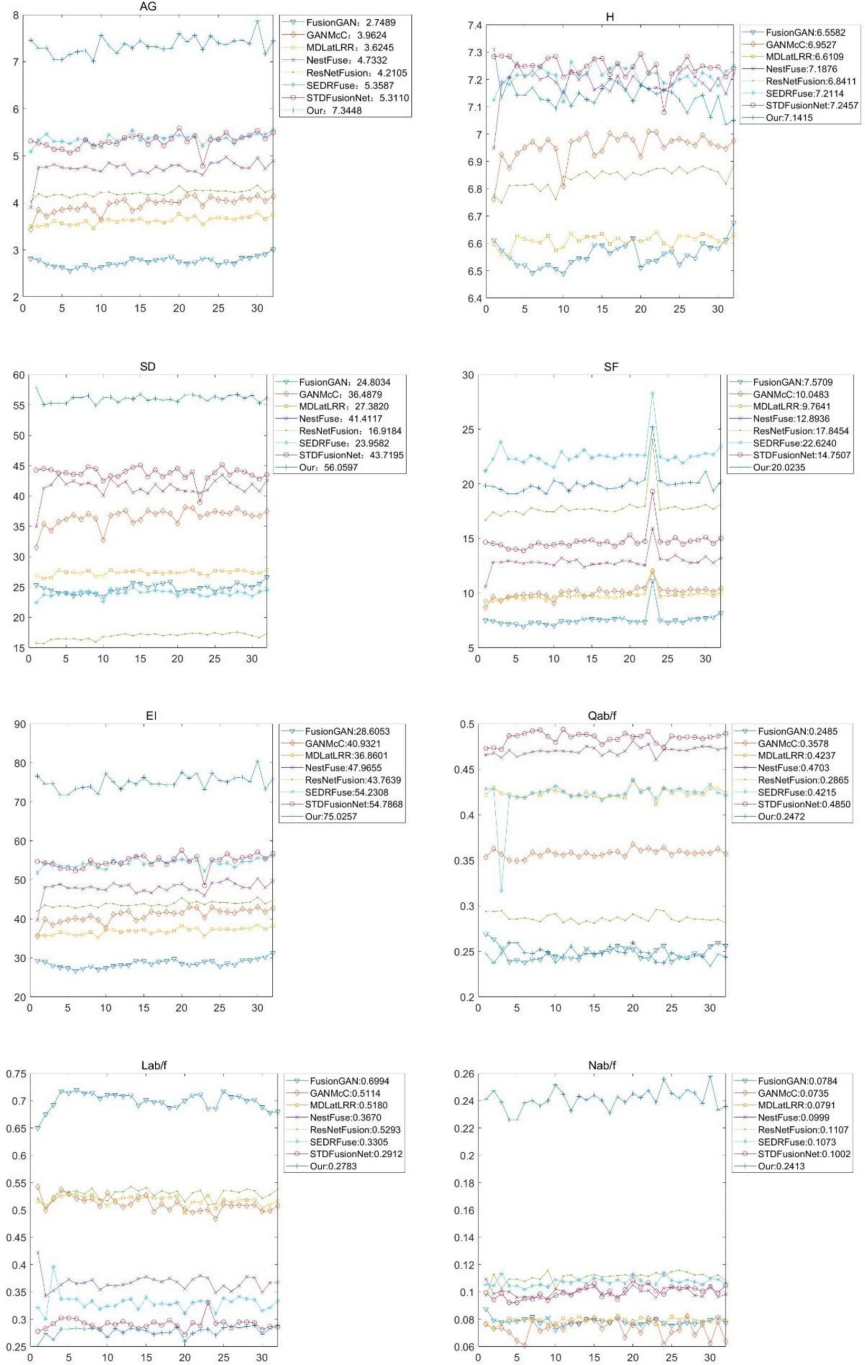
Quantitative comparisons in terms of the eight metrics; AG, H, SD, SF, EI, Q^ab/f^, L^ab/f^, and N^ab/f^, for the Nato_campsequence from the TNO dataset.

As shown in Figs 10–12, all the methods provide comparable fusion results with respective advantages. In overall quality, FusionGAN, GANMcC, and ResNetFusion generate more infrared-like images, taking advantage of a significant target retaining heat source but losing the background texture information. Although the MDLatLRR algorithm can keep the heat source target and background texture information at the same time, the overall fusion result is fuzzy, the significance of the heat source target is lost, and the target is not easily found. NestFuse, SEDRFuse, STDFusionNet, and our method can clearly reflect the critical information in infrared and visible images. In combination with the visible light evaluation analysis, our results of AG, SD, and EI evaluation indexes ranked first in all the images except for the fifth image (third rank). In terms of the H and SF indices, our method ranked second, reflecting the change degree between image pixels and the sharpness of the image. From the perspective of the evaluation indicators, the sharpness of this paper is superior to the other compared methods in most of the evaluation indicators. To reflect the fusion image consisting of infrared and visible light image information of each evaluation function Q^ab\f^, the mean of the evaluation results of our algorithm is 0.3505, which is at the median level, showing that our results fusion of infrared and visible light information is not the most. The reason is that the infrared image contains a lot of interference information, such as heat-source target (e.g., humans), which makes the background saturated. Also, over-exposed backgrounds in visible light images influence the observation of the background texture. The proposed method can adaptively compensate for this part of the interference, obtaining a clearly fused image. From the loss function L^ab/f^ and artifact function N^ab/f^, it can be proved that although some information is lost in our algorithm, both of them are loss interference information. Therefore, in Lab/f evaluation, our results can provide the best performance, and N^ab/f^ evaluation value is the largest. It also proves that our algorithm considers the average of infrared and visible image information. When some parts where heat-source target highlights, our fusion results can also be observed that the texture of the goals of the heat source form, N^ab/f^ large source of value, i.e., the cause of the picture without the part information. Our algorithm uses adaptive weights to jointly display the information of infrared and visible images in this area in order to make full use of the fusion advantages; thus, the average N^ab/f^ is too large.

**Fig 13:**
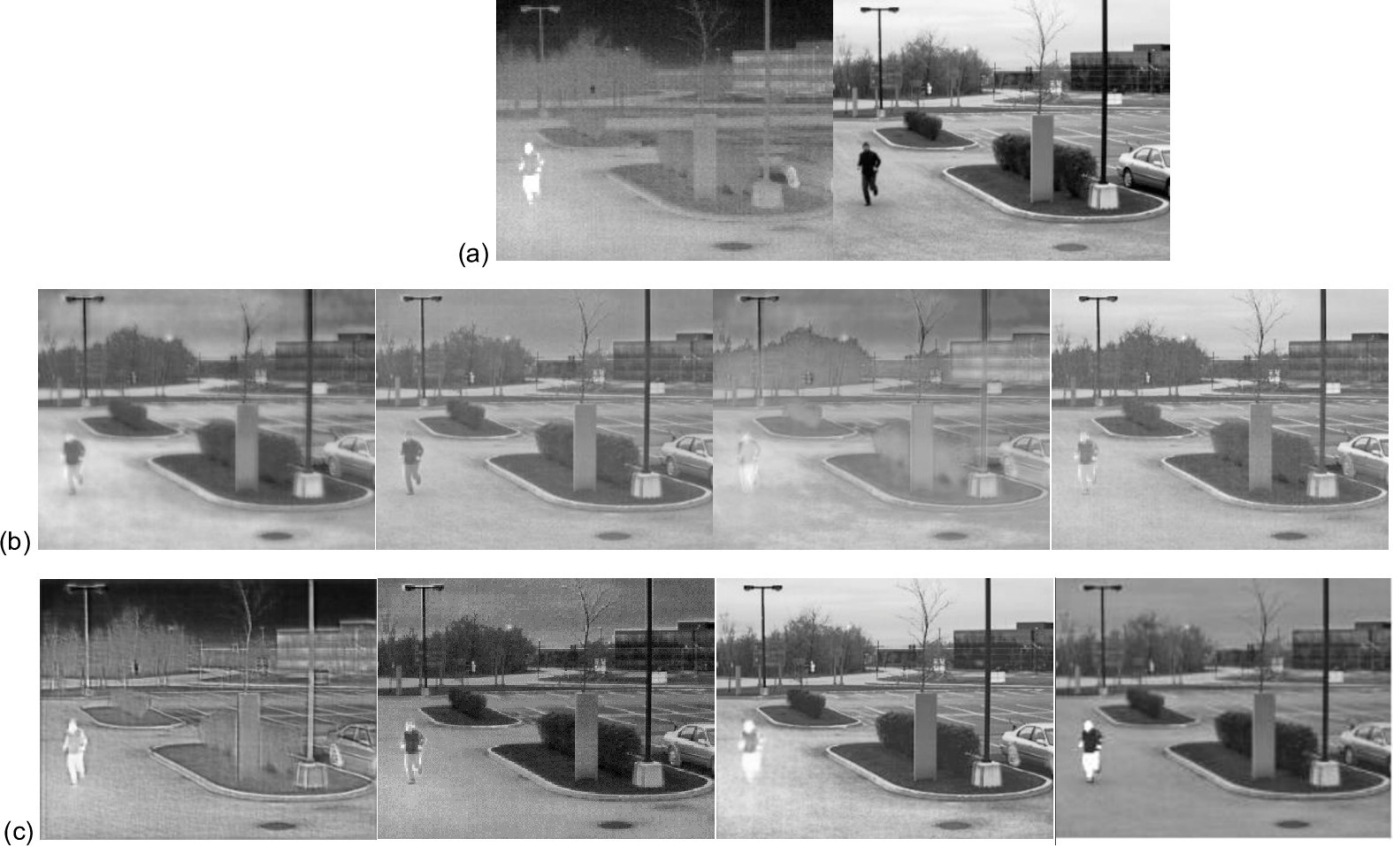
Qualitative comparison for 20th frame of Trees and runner from the INO dataset. (a) the infrared and visible images.(b) the fusion results of FusionGAN, GANMcC, MDLatLRR, and NestFuse. *(c)* the fusion results of ResNetFusion, SEDRFuse, STDFusionNet, and OUR.

**Fig 14:**
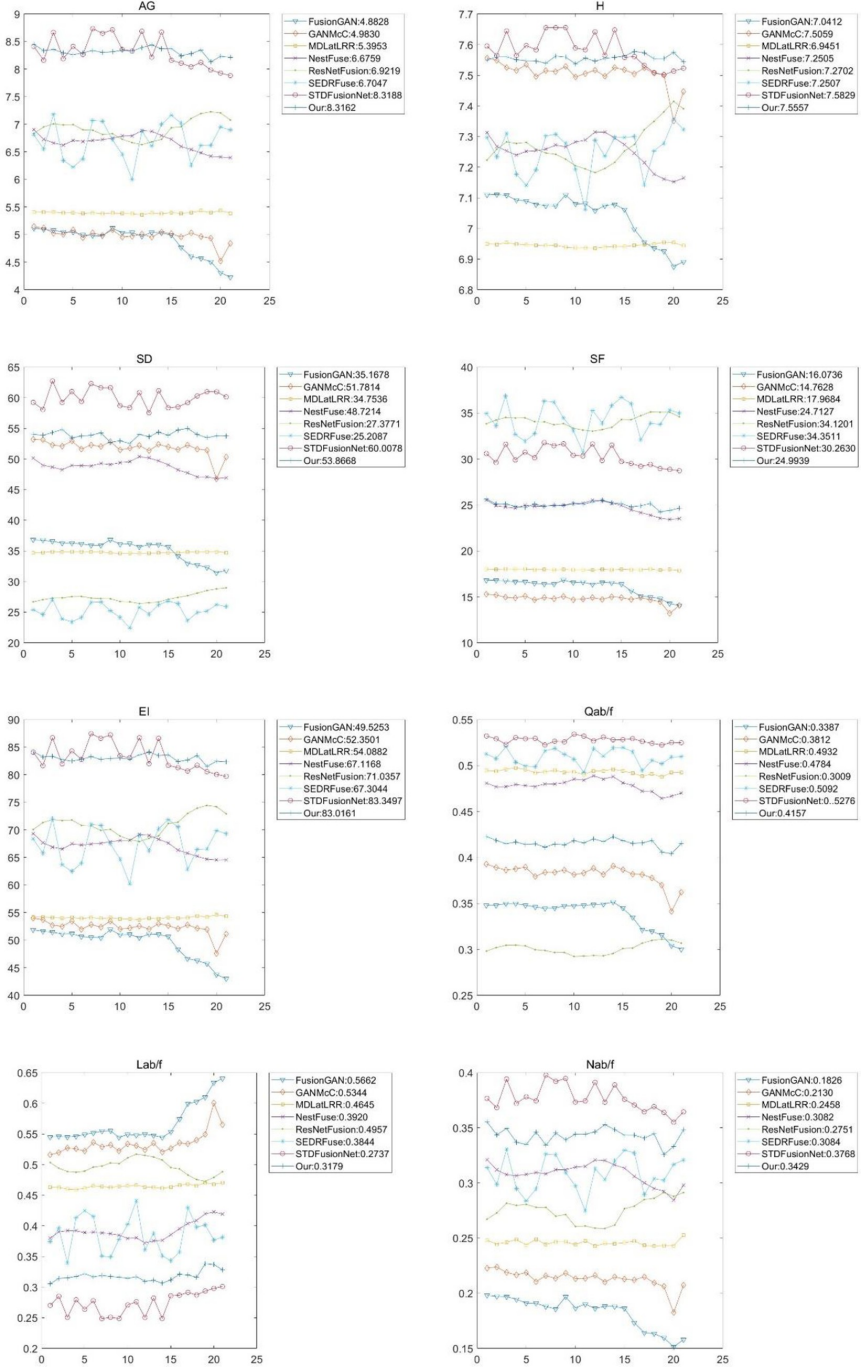
Quantitative comparisons in terms of the eight metrics, AG, H, SD, SF, EI, Q ^ab/f^, L^ab/f^, and N^ab/f^, for the Nato_campsequence from the Trees and runner sequence from the INO dataset.

The compared methods were also evaluated on different weather conditions of images from the INO dataset. The videos, Runner and Tree, were split into 21 image frames, which were used for quantitative and qualitative evaluations. Figs 13 and 14 present the qualitative and quantitative comparisons, respectively, showing that all the eight compared algorithms can preserve texture information well. However, FusionGAN and ResNetFusion generated infrared-lie results, while GANMcC generated a relatively fuzzy result. NestFuse, SEDRFUSE, and STDFusionNet can retain visible light texture and infrared heat source targets. Our algorithm can retain not only infrared information but also background texture information with better sharpness. As quantitative evaluations show, our algorithm also can provide high robustness, clarity, and balanced information inclusion between infrared and visible images.

It should be noted that in practical engineering, the operation time of the algorithm is critical. Since the proposed algorithm operates pixel-wise in the spatial domain, the computational complexity is far lower than other frequency-domain algorithms. Table 2 compares the computational time for each dataset.

**Table 2:** Computational time comparison. All the experiments were performed on the CPU. (unit: s).

Method	TNO1	TNO2	TNO3
FusionGAN	**4.25**	1.24	1.04
GANMcC	9.12	2.42	2.17
MDLatLRR	3.14 x 10^1^	9.20	7.82
NestFuse	1.84 x 10^1^	5.36	4.55
ResNetFusion	1.87	5.45 x 10^−1^	4.73 x 10^−1^
SEDRFuse	1.24 x 10^1^	4.61	4.11
STDFusionNet	1.36	4.05 x 10^−1^	3.33 x 10^−1^
Our	**7.99 x 10** ^**−1**^	**2.72 x 10** ^**−1**^	**2.38 x 10** ^**−1**^

## Conclusion

This study employs the clustering algorithm to decompose the source image into the spatial domain directly. The results indicate that the multi-scale image decomposition into the spatial domain can extract more image features, eliminating the requirements while converting from the frequency domain to the spatial domain. As a result, the proposed algorithm consumes less time than the contrasting algorithm, and generates fusion results with higher clarity. Although the image features of the layers are employed as a reference for the fusion weights in the fusion process of the extracted layers, from a global perspective, these weights are still relatively rough. At the same time, feature extraction relies on the internal information of the source image, and there is a lack of correlation between the source image and the fused one, resulting in a poor fit of the proposed feature layer. When extracting image features, each source image can reference each other so that the obtained layer features have a high degree of fit. In future research, a new multi-scale tool will be applied to solve this problem. At the same time, the fusion weights with better performance are further explored.
